# New cytotoxic benzo(b)thiophenilsulfonamide 1,1-dioxide derivatives inhibit a NADH oxidase located in plasma membranes of tumour cells

**DOI:** 10.1054/bjoc.2001.2083

**Published:** 2001-09-01

**Authors:** M M Alonso, I Encío, V Martínez-Merino, M Gil, M Migliaccio

**Affiliations:** Department of Health Sciences, Public University of Navarra, Avd Barañaín s/n, Pamplona, 31008, Spain; Department of Applied Chemistry, Public University of Navarra, Campus de Arrosadía, Pamplona, 31006, Spain

**Keywords:** diarylsulfonylureas, antineoplasic drugs, NADH oxidase, CCRF-CEM, plasma membrane

## Abstract

A series of benzo(b)thiophenesulfonamide 1,1-dioxide derivatives (BTS) have been designed and synthesized as candidate antineoplastic drugs. Several of these compounds have shown in vitro cytotoxic activity on leukaemic CCRF-CEM cells. The cytotoxic BTS, but not the inactive ones, were able to inhibit a tumour cell-specific NADH oxidase activity present in the membrane of CCRF-CEM cells. © 2001 Cancer Research Campaign


*British Journal of Cancer* (2001) **85**(9), 1400–1402
© 2001 Cancer Research Campaign
doi: 10.1054/ bjoc.2001.2083, available online at http://www.idealibrary.com on IDEAL

http://www.bjcancer.com

Short Communication

### New cytotoxic benzo(b)thiophenilsulfonamide 1,1-dioxide derivatives inhibit a NADH oxidase located in plasma membranes of tumour cells

**MM Alonso[1], I Encío[1], V Martínez-Merino[2], M Gil[2] and M Migliaccio[1]**

[1]Department of Health Sciences, Public University of Navarra. Avd Barañaín s/n, 31008 Pamplona, Spain; [2]Department of Applied Chemistry, Public University of Navarra. Campus de Arrosadía 31006 Pamplona, Spain

**Summary** A series of benzo(b)thiophenesulfonamide 1,1-dioxide derivatives (BTS) have been designed and synthesized as candidate antineoplastic drugs. Several of these compounds have shown in vitro cytotoxic activity on leukaemic CCRF-CEM cells. The cytotoxic BTS, but not the inactive ones, were able to inhibit a tumour cell-specific NADH oxidase activity present in the membrane of CCRF-CEM cells. © 2001 Cancer Research Campaign

**Keywords**: diarylsulfonylureas; antineoplasic drugs; NADH oxidase; CCRF-CEM; plasma membrane

Some compounds belonging to the family of the diarylsulfonylureas (DSU) show significant antitumour activity in in vitro and in vivo assays (Howbert et al, 1990; Houghton and Houghton, 1996). One of the main interests of DSU is that the mechanism by which they exert their toxic effect on tumour cells remains unknown, and seems to be different from the mechanisms described for other antitumour cytotoxic drugs (Sosinski et al, 1993). Until now, the only putative cellular target that has been proposed for these compounds is a protein of 34 kD with a NADH oxidase activity that can be inhibited by thiol reagents such as the N-ethyl-maleimide (NEM). This protein is associated with the membranes of tumour cells and is also found in the serum of cancer patients but not in that of normal donors (Kim et al, 1997). Morré et al have demonstrated that the active sulfonylurea LY181984 binds to and inhibits the activity of this enzyme (Morre et al, 1995b,d). On the basis of theoretic structure-activity COMFA studies, we designed, synthesized and tested in in vitro antitumour assays several analogues and derivatives of DSU (Gil et al, 1999; Martínez-Merino et al, 2000) in the hope of finding new compounds with improved antitumoural activity and reduced toxicity. Based on the results obtained in that work we have designed and synthesized several benzo(b)thiophenesulfonamide 1,1-dioxide derivatives (BTS) some of which are structurally related with DSU.

Here we describe the effect of 6 BTS on a NADH oxidase activity found in plasma membranes and conditioned culture medium of CCRF-CEM cells whose kinetic properties correspond to those of the protein described by Morré et al, in other tumour cells as a putative target of DSU.

*Received 24 April 2001*
*Revised 3 July 2001*
*Accepted 18 July 2001*

*Correspondence to:* M Migliaccio

### MATERIALS AND METHODS

### Cell culture

CCRF-CEM cells (ATCC, Manassas, VA) were grown in RPMI 1640 medium (Gibco, Grand Island, NY) supplemented with 10% fetal calf serum, glutamine (2 mM) and gentamicyn (0.1 mg $ml^{-1}$). Primary rat vascular smooth muscle cells were isolated and cultured according to Bacakova et al (1997).

### Candidate antineoplastic drugs

The compounds used in this work were synthesized as described (Martínez-Merino et al, 2000). Candidate drugs were initially dissolved in DMSO at 0.1 M, and then diluted with complete culture medium to the final concentrations indicated in every assay.

### Cytotoxicity assay

CCRF-CEM cells were cultured in the presence of the compounds to be tested (100 μM) for the indicated periods of time. The cytotoxic effect was determined by following spectrophotometrically the release of cytoplasmic lactate dehydrogenase (LDH) to the culture medium with a Cytotoxicity Detection Kit (Roche, Indianapolis, IN). The percentage of cytotoxicity was calculated as follows: [(experimental release – spontaneous release)/(maximum release – spontaneous release)] × 100. Spontaneous release and maximum release were obtained by incubating the cells alone or with a 0.1% Triton x-100 solution respectively.

### NADH oxidase activity measurement

NADH oxidase activity was studied in samples of isolated plasma membranes and culture supernatants of CCRF-CEM cells following the method described by Morré et al (1995c) with minor modifications. Plasma membranes were isolated by aqueous 2-phase partition (Morre et al, 1995c). Conditioned medium was obtained from 24 hour cultures (initial cell density of $10^6$ cells $ml^{-1}$) by centrifugation for 20 minutes at 2500 rpm. NADH oxidase activity was determined at 37˚C as the disappearance of NADH measured at 340 nm in a reaction mixture containing 25 mM Tris-Mes buffer (pH 7.2), 1 mM KCN and the indicated concentrations of NADH (Sigma, St Louis, MO). Absorbance was recorded 30 seconds and 5 minutes after adding NADH, and a millimolar extinction coefficient of 6.22 was used to calculate the rate of NADH disappearance. Compounds to be tested as inhibitors were added at final concentrations ranging from 100 to 0.1 μM. Percentages of inhibition were calculated by the formula: [1-(enzymatic activity in the presence of inhibitor/enzymatic activity in the absence of inhibitor)] × 100.

### RESULTS

The structure of BTS included in this study is shown in Figure 1. We chose the acute lymphoblastic T leukaemia cell line CCRF-CEM as a model to investigate the activity of these compounds on tumour cells for 2 main reasons. First, because it has been used for the screening of many candidate antitumour drugs, including a high number of DSU (Howbert et al, 1990), and therefore the use of this cell line allows us to compare our results with those data. Second, because these cells have been also used to characterize with some detail the process of cell death in response to different antineoplasic drugs (Huschtscha et al, 1996). To test the cytotoxic activity of BTS compounds on CCRF-CEM cells we performed a series of in vitro cytotoxicity assays as described in Materials and methods. As shown in Figure 2, all tested BTS except the compound D displayed a clear cytotoxic activity on CCRF-CEM cells that is basically completed 48 hours after drug addition. As we said before, until now the only cell target proposed for DSU is a tumour-specific NADH oxidase enzyme described by Morré et al in HeLa and other tumour, whose activity can be inhibited by DSU and thiol reagents (Morre et al, 1995a,c,d). As the BTS used in this work were designed on the basis of experimental results obtained with DSU, and some BTS are structurally closely related with them, it appeared interesting to investigate if these compounds also exert some effect in this NADH oxidase.

As a first step we performed some experiments in order to investigate if a thiol reagent-sensitive NADH oxidase activity could be detected in CCRF-CEM cell membranes and supernatants. As shown in Figures 3A and B, a clear activity could be detected in both CCRF-CEM membranes and conditioned culture medium, respectively. The $K_M$ values obtained in both cases were basically identical (27 and 28 μM), and coincide with those obtained by Morré et al in membranes and culture supernatants from other tumour cells (Morre et al, 1995d). Moreover, thiol reagents such as NEM can inhibit this activity (data not shown). All these data strongly suggest that the enzyme responsible for this activity in samples from CCRF-CEM cells corresponds to that previously described by Morré et al in other tumour cells. It has been previously reported that this thiol reagent-sensitive NADH activity is specific of tumour cells and not detectable in normal cells (Morre et al, 1995a,d). In agreement with that, we failed to detect this activity in conditioned culture medium from normal rat smooth muscle cells (data not shown). As reported for other primary normal cells (Morre et al, 1995a,d), only a thiol reagent-resistant, non tumour-specific NADH oxidase activity was detected in smooth muscle cell supernatants.

Finally, to test if the newly synthesized compounds were able to inhibit the NADH oxidase activity characterized in CCRF-CEM cells, enzymatic assays were repeated with samples of plasma membranes and culture supernatants in the presence of NADH 0.15 mM and all the compounds investigated at doses ranging from 0.1 to 100 μM. As depicted in Figures 4A and B, the 5 compounds that previously showed cytotoxic activity on CCRF-CEM cells

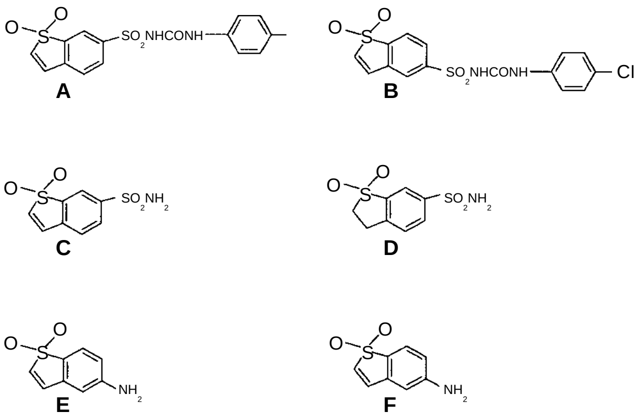

**Figure 1** Structure of BTS used in this work

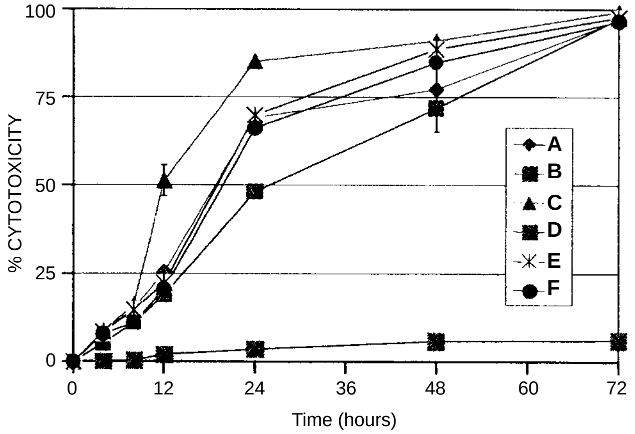

**Figure 2** Determination of the cytotoxic effect of 6 BTS on CCRF-CEM cells. Cells were incubated in the presence of every compound (100 μM) for the indicated periods of time. Culture supernatants were collected and cell death quantification was performed by the measurement of the LDH enzymatic activity released from damaged cells to the culture medium, as described in material and methods. Values represent means ±SD derived from 3 independent experiments each performed in quadruplicate

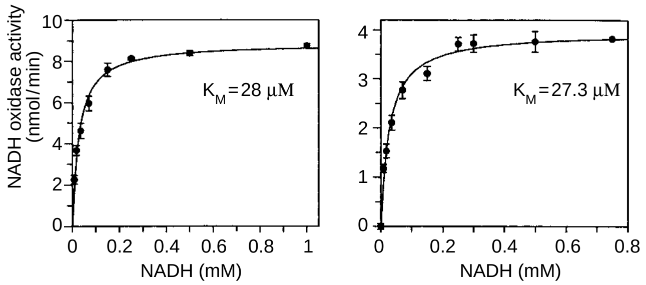

**Figure 3** Kinetics of NADH oxidase activity found in membrane extracts (**A**) and conditioned cultured medium (**B**) of CCRF-CEM cells. The insets show the calculated $K_M$ values. Means ± SD derived from 3 independent experiments each performed in triplicate are presented

*© 2001 Cancer Research Campaign* 

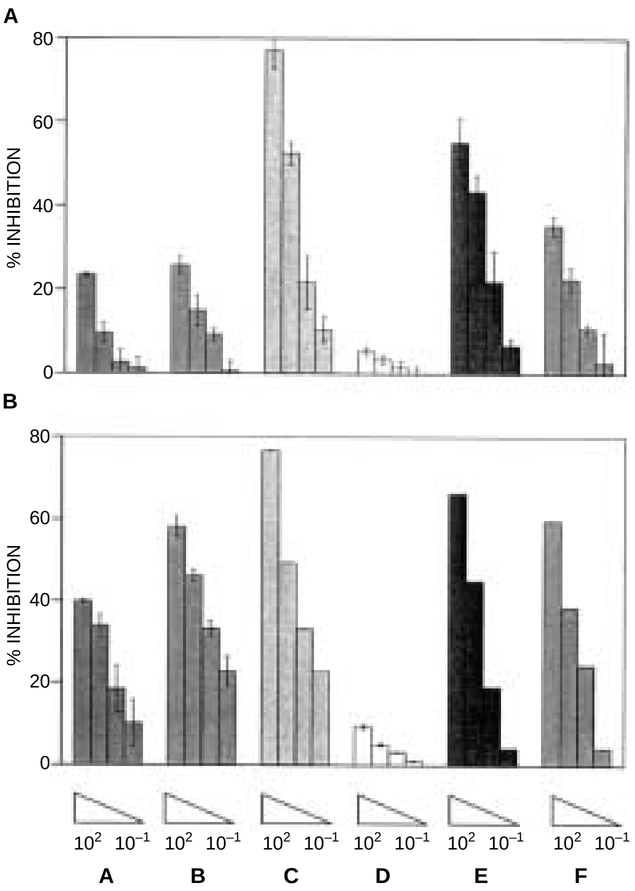

**Figure 4** BTS inhibits the tumour-specific NADH oxidase activity of CCRF-CEM cell membranes (**A**) and culture supernatants (**B**). NADH oxidase activity in samples of membrane extracts and culture supernatants in the presence of NADH (0.15 mM) and BTS derivatives (100, 10.1 and 0.1 μm) was determined as described in Materials and methods. Percentages of inhibition were calculated as described in Materials and methods. Columns and error bars represent means and standard deviations from 3 independent experiments each performed in triplicate

(Figure 2), also inhibited in a dose-dependent fashion the NADH oxidase studied. The levels of inhibition reached at 100 μM were similar or higher than those obtained with NEM, indicating that the residual activity corresponds to the non-tumour-specific, thiol reagent-resistant activity also found in normal cells. Interestingly, compound D, that is the reduced derivative of compound C and showed no cytotoxicity on CCRF-CEM cells, also lacked any inhibitory activity on the enzymatic assay, suggesting that both effects could be related.

### DISCUSSION

In this work we described the effect of new BTS derivatives in leukaemic CCRF-CEM cells. We found a clear correlation between the cytotoxic effect of the compounds tested and their ability to inhibit an NADH oxidase located in the membrane of these and other tumour cells. It is very significant that the compound D, structurally very close to other compounds tested, was inactive both in the cytotoxicity and the enzymatic assays. These data suggest that the inhibition of this enzyme might play a role in the cytotoxic effect of the tested BTS. The described NADH oxidase activity most probably corresponds to the one previously described by Morré et al (1995c). This enzyme, whose physiologic function is unknown, has been proposed to be the target of DSU (Morre et al, 1995d) and other cytotoxic drugs such as capsaicin (Morre et al, 1995a), adriamicyn (Morre et al, 1997) and anticancer quassinoids (Morre et al, 1998). The elucidation of the function of this enzyme remains as the key to understand its role in the effect of BTS and other cytotoxic drugs.

### REFERENCES

Bacakova L, Mares V, Lisa V and Kocourek F (1997) Sex-dependent differences in growth and morphology of cultured vascular smooth muscle cells from newborn rats. *Physiol Res* **46**: 403–406

Gil MJ, Manu MA, Arteaga C, Migliaccio M, Encio I, Gonzalez A and Martinez-Merino V (1999) Synthesis and cytotoxic activity of N-(2-pyridylsulfenyl)urea derivatives. A new class of potential antineoplastic agents. *Bioorg Med Chem Lett* **9**: 2321–2324

Howbert JJ, Grossman CS, Crowell TA, Rieder BJ, Harper RW, Kramer KE, Tao EV, Aikins J, Poore GA, Rinzel SM et al (1990) Novel agents effective against solid tumors: the diarylsulfonylureas. Synthesis, activities, and analysis of quantitative structure-activity relationships. *J Med Chem* **33**: 2393–2407

Houghton PJ and Houghton JA (1996) Antitumor diarylsulfonylureas: novel agents with unfulfilled promise. *Invest New Drugs* **14**(3): 271–280

Huschtscha LI, Bartier WA, Ross CE and Tattersall MH (1996) Characteristics of cancer cell death after exposure to cytotoxic drugs in vitro. *Br J Cancer* **73**: 54–60

Kim C, MacKellar WC, Cho NM, Byrn SR and Morre DJ (1997) Impermeant antitumor sulfonylurea conjugates that inhibit plasma membrane NADH oxidase and growth of HeLa cells in culture. Identification of binding proteins from sera of cancer patients. *Biochim Biophys Acta* **1324**: 171–181

Martinez-Merino V, Gil MJ, Encío I, Migliaccio M and Arteaaga C (2000) Benzo[b]thiophene sulfonamide-1, 1-dioxido derivatives and their use as antineoplastic agents. Patent N° WO 00/63202

Morre DJ, Chueh PJ and Morre DM (1995a) Capsaicin inhibits preferentially the NADH oxidase and growth of transformed cells in culture. *Proc Natl Acad Sci USA* **92**: 1831–1835

Morre DJ, Morre DM, Stevenson J, MacKellar W and McClure D (1995b) HeLa plasma membranes bind the antitumor sulfonylurea LY181984 with high affinity. *Biochim Biophys Acta* **1244**: 133–140

Morre DJ, Wilkinson FE, Lawrence J, Cho N and Paulik M (1995c) Identification of antitumor sulfonylurea binding proteins of HeLa plasma membranes. *Biochim Biophys Acta* **1236**: 237–243

Morre DJ, Wu LY and Morre DM (1995d) The antitumor sulfonylurea N-(4-methylphenylsulfonyl)-N′-(4-chlorophenyl) urea (LY181984) inhibits NADH oxidase activity of HeLa plasma membranes. *Biochim Biophys Acta* **1240**: 11–17

Morre DJ, Kim C, Paulik M, Morre DM and Faulk WP (1997) Is the drug-responsive NADH oxidase of the cancer cell plasma membrane a molecular target for adriamycin? *J Bioenerg Biomembr* **29**: 269–280

Morre DJ, Grieco PA and Morre DM (1998) Mode of action of the anticancer quassinoids–inhibition of the plasma membrane NADH oxidase. *Life Sci* **63**: 595–604

Sosinski J, Thakar JH, Germain GS, Harwood FC and Houghton PJ (1993) Proliferation-dependent and -independent cytotoxicity by antitumor diarylsulfonylureas. Indication of multiple mechanisms of drug action. *Biochem Pharmacol* **45**: 2135–2142

*© 2001 Cancer Research Campaign*

